# Approach to prolactin monitoring and hyperprolactinaemia in transgender and gender-diverse individuals undergoing gender affirming hormone therapy

**DOI:** 10.3389/fendo.2025.1608108

**Published:** 2025-05-27

**Authors:** Brendan J. Nolan, Matthew I. Accatino, Lachlan M. Angus, Ada S. Cheung

**Affiliations:** ^1^ Trans Health Research Group, Department of Medicine (Austin Health), University of Melbourne, Heidelberg, Victoria, Australia; ^2^ Department of Diabetes and Endocrinology, Princess Alexandra Hospital, Woolloongabba, Queensland, Australia; ^3^ Faculty of Medicine, University of Queensland, Brisbane, Queensland, Australia; ^4^ Royal Brisbane and Women’s Hospital, Herston, Queensland, Australia; ^5^ Northern Adelaide Local Health Network, Elizabeth Vale, South Australia, Australia; ^6^ Department of Endocrinology, Austin Health, Heidelberg, Victoria, Australia

**Keywords:** transgender, prolactin, estradiol, cyproterone acetate (CPA), gender affirming hormonal therapy (GAHT)

## 1 Introduction

Transgender and gender-diverse (herein, trans) individuals who desire feminisation are typically treated with estradiol and an anti-androgen such as spironolactone or cyproterone acetate (1–3). This results in development of physical changes which align an individual’s physical appearance with their gender identity and improves psychological functioning (4, 5).

Consensus guidelines provide recommendations for sex steroid monitoring of serum estradiol and testosterone concentrations for individuals undergoing gender affirming hormone therapy (GAHT). Given the knowledge that estrogens can stimulate growth of lactotroph cells and case reports of prolactinoma in trans individuals treated with feminising GAHT, some guidelines recommend monitoring serum prolactin concentration (1, 6). Notably, there are sex-specific serum prolactin reference ranges in the general population, with reference intervals higher in the female population. It should also be noted that the initial observations of hyperprolactinaemia and prolactinomas were in individuals prescribed estrogen formulations and cyproterone acetate doses that are no longer recommended (7, 8), so the utility of routine prolactin monitoring must be weighed against potentially unnecessary investigations and associated healthcare costs.

There are two unanswered questions. Firstly, should clinicians routinely monitor prolactin in trans individuals undergoing feminising GAHT? Secondly, how should clinicians approach investigation and management of trans individuals with symptomatic hyperprolactinaemia? We herein review the current evidence and provide a suggested approach to the investigation and management of symptomatic hyperprolactinaemia in trans individuals undergoing feminising GAHT.

### 1.1 Causes of hyperprolactinaemia

The differential diagnosis of hyperprolactinaemia includes prolactinoma, macroprolactinaemia, psychoactive medications, estrogen therapy, renal failure, and hypothyroidism (9, 10). Most patients with serum prolactin concentrations >3000 mIU/L will have a prolactinoma (11, 12); however, medications including metoclopramide, risperidone, and phenothiazines may result in prolactin concentrations above this range (13). Medication dose is another important consideration, such as a dose-dependent change in serum prolactin with conjugated equine estrogens and ethinylestradiol in cisgender women (8, 14) and cyproterone acetate in trans women (15).

## 2 What is the impact of feminising GAHT on serum prolactin concentrations?

### 2.1 Cyproterone acetate

The association between cyproterone acetate and hyperprolactinaemia is well-established (16, 17). Proposed mechanisms for hyperprolactinaemia with cyproterone acetate include progestogenic activity and/or a reduction in hypothalamic dopamine (18). In a prospective cohort of 61 trans women in the Netherlands, serum prolactin increased from 150 mIU/L at baseline to 440 mIU/L at month 3, with a nonsignificant change from month 3 to 12 (16). In this analysis, 9 (15%) individuals had prolactin above the upper limit of the reference interval (600 mIU/L) at month 3 and 18 (30%) at month 12. The authors also undertook a retrospective evaluation of 38 trans women who had undergone gonadectomy. In this cohort, serum prolactin increased following during GAHT (mean prolactin 360 mIU/L) but returned to baseline following cessation of cyproterone acetate post gonadectomy (prolactin 160 mIU/L) (16). No individuals in this study developed a serum prolactin concentration >2000 mIU/L. Similar results were demonstrated in another prospective evaluation from Belgium. In this study, 107 trans women initiating estradiol and cyproterone acetate had serum prolactin measurements at baseline, pre- and post-gonadectomy, or at baseline, month 12 and month 18 for those who did not undergo gonadectomy. For individuals who underwent gonadectomy, mean baseline prolactin was 200 mIU/L, increased to 505 mIU/L during GAHT, and fell back to 216 mIU/L post gonadectomy (19). In the group who did not undergo gonadectomy, mean baseline serum prolactin measured 211 mIU/L, increased to 490 mIU/L after 12 months of GAHT, and remained at 300 mIU/L after 18 months of GAHT. Notably, all individuals in these studies were treated with cyproterone acetate 50mg daily which is no longer recommended.

Cyproterone acetate dose appears to influence the degree of hyperprolactinaemia. A 12-month prospective cohort study of 882 trans individuals found an association between higher cyproterone doses and higher serum prolactin concentration (15). In this analysis, hyperprolactinaemia (prolactin >600 mIU/L) was not reported in individuals treated with estradiol without cyproterone acetate. However, hyperprolactinaemia was documented in 9.1% of individuals using cyproterone acetate 10mg daily, 11.7% using 25mg daily, 13.8% using 50mg daily and 14.3% using 100mg daily. Similarly, in a retrospective cohort study, individuals using low-dose (n=38, mean CPA dose 11.6 ± 3.7mg daily) were compared to a high-dose group (n=26, mean CPA dose 61.5 ± 21.5mg). Both groups had an increase in serum prolactin concentration over 12 months but this remained higher in the high-dose group at 12 months, after adjusting for serum estradiol concentration (20).

### 2.2 Cyproterone acetate compared to spironolactone or gonadotropin releasing hormone analogues

Studies have consistently demonstrated higher serum prolactin concentrations with cyproterone acetate compared to spironolactone or gonadotropin releasing hormone (GnRH) analogues.

Randomised trials comparing cyproterone acetate and spironolactone as a component of feminising GAHT regimens demonstrate higher serum prolactin concentrations with cyproterone acetate (21, 22). Importantly, serum prolactin remained below twice the upper limit of the reference interval over 6 months on cyproterone acetate 12.5mg daily (21). Similar findings have been documented in retrospective studies (23, 24). In another analysis from the United States, treatment with estradiol and spironolactone was not associated with an increase in prolactin or hyperprolactinaemia (25).

Cyproterone acetate has also been associated with higher serum prolactin concentrations in retrospective studies with comparator groups treated with GnRH analogues (26, 27). Over 12 months, one study reported an increase in serum prolactin with cyproterone acetate (237 ± 156 mIU/L at baseline vs. 574 ± 241 mIU/L at 12 months) but not leuprolide acetate (260 ± 125 vs. 313 ± 142) (26). Only 1 (5%) individual treated with cyproterone acetate developed hyperprolactinaemia. Similarly, another retrospective study documented higher serum prolactin concentrations in people treated with cyproterone acetate compared to spironolactone or GnRH analogues (28).

### 2.3 Reference intervals for serum prolactin for individuals established on feminising GAHT

A large cohort study from the Netherlands determined serum prolactin reference intervals (2.5-97.5^th^ centile) in trans women prior to initiation of GAHT, approximately 1 year after GAHT, and following gonadectomy (29). It should be noted that cyproterone acetate 50-100mg daily was prescribed in this cohort, which was discontinued after gonadectomy. Serum prolactin increased following initiation of GAHT (reference interval, 100–1020 mIU/L) and decreased after gonadectomy (70–720 mIU/L), though remained higher than baseline (50–310 mIU/L) (29). Another cross-sectional analysis from the United States included 93 trans women established on feminising GAHT for at least one year (30). The prolactin reference interval in this cohort was found to be 70–468 mIU/L and 104–680 mIU/L using the Beckman Coulter DxI and Roche Cobas immunoassay, respectively (30). In this cohort, 39 (42%) were treated with spironolactone but none were treated with cyproterone acetate. In the absence of a locally validated reference interval in the trans population, we therefore suggest use of the female reference interval as this more closely approximates that of trans people established on feminising GAHT (29). As the laboratory typically reports one sex-specific reference interval for each analyte, clinicians should check the reference interval that is reported and consider specifying reporting of the female prolactin reference interval (31).

## 3 What is the risk of prolactinoma in individuals treated with feminising GAHT?

There are limited data evaluating the risk of prolactinoma in trans people undergoing feminising GAHT. The largest series reported 9 prolactinomas from a cohort of 2555 trans women, of which 5 were symptomatic [galactorrhoea (n=4) and hypothyroidism (n=1)] (32). The authors calculated standardised incidence ratios (SIRs) compared to expected cases in the general population, and found a higher SIR compared to the general female population (SIR, 4.3 (2.1-7.9) (32). However, this was not different to the general population when only the symptomatic prolactinomas were included [SIR, 2.4 (0.9-5.3)]. Serum prolactin concentration was not reported in this analysis. A previous systematic review of 8 patients with a prolactinoma noted serum prolactin concentration 2300 mIU/L or higher in all individuals, with 6 having serum prolactin concentration >3000 mIU/L (33). Serum prolactin concentration exceeded this value in a subsequent case series including a further 2 individuals with prolactinomas during treatment with feminising GAHT (34).

## 4 Should clinicians routinely monitor prolactin in trans individuals treated with feminising GAHT?

Hyperprolactinaemia is a potential adverse effect of feminising GAHT. Given this, current Endocrine Society guidelines suggest monitoring serum prolactin concentration at baseline, annually during the transition period and then every 2 years thereafter (1), whereas Italian guidelines suggest monitoring serum prolactin periodically (35). However, some clinicians do not monitor prolactin unless there are symptoms of hyperprolactinaemia such as galactorrhoea or visual field disturbance (36, 37). Reflecting this uncertainty, the 2022 World Professional Association for Transgender Health Standards of Care Version 8 (SOC-8) did not give a recommendation regarding prolactin monitoring, but instead suggested individualised clinical decision making based on the GAHT regimen and/or the presence of symptoms of hyperprolactinaemia or pituitary tumour (2).

## 5 How should clinicians approach investigation and management in trans individuals with symptomatic hyperprolactinaemia?

Given the associations between feminising GAHT and mild hyperprolactinaemia, we advocate against the routine monitoring of prolactin unless there are symptoms suggestive of hyperprolactinaemia. This pragmatic approach aims to minimise potentially unnecessary investigations including pituitary MRI (magnetic resonance imaging), associated healthcare costs and patient anxiety.

**Figure 1:**
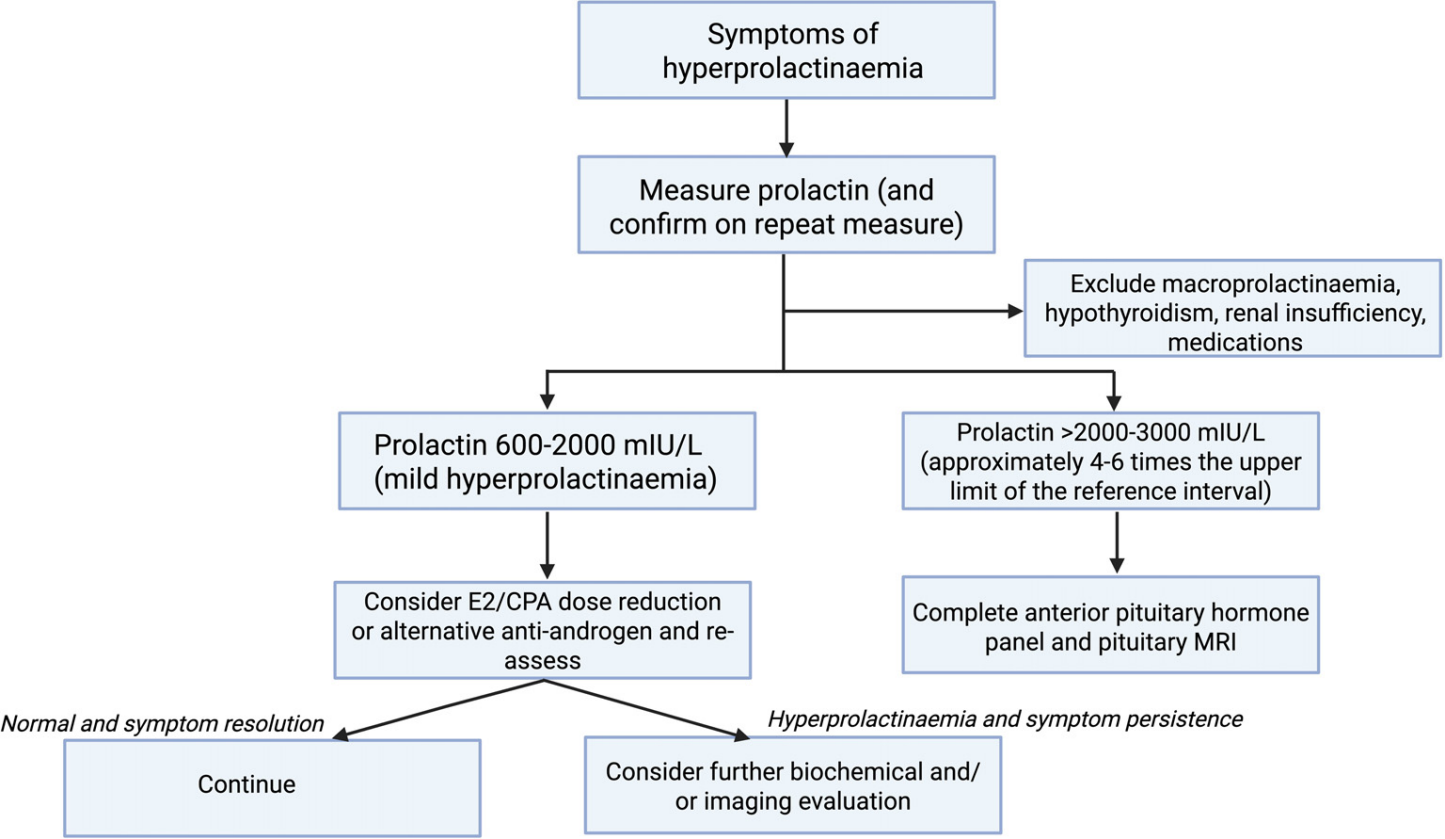
Suggested algorithm for investigation and management of symptomatic hyperprolactinaemia in trans individuals undergoing feminising GAHT. Created in BioRender. Nolan, B. (2025) https://BioRender.com/r68y190.

**Figure 1** demonstrates a suggested algorithm for the investigation and management of symptomatic hyperprolactinaemia in trans individuals undergoing feminising GAHT. We suggest first confirming hyperprolactinaemia on repeat testing and exclusion of other causes of hyperprolactinaemia. Notably, the trans population have high rates of psychotropic medication prescription, which can increase serum prolactin (38, 39). A serum prolactin concentration cut-off of >2000–3000 mIU/L (or approximately 4–6 times the upper limit of the reference interval) could be used to guide investigations including anterior pituitary hormone panel and pituitary MRI, given that most prolactinomas will have serum prolactin concentration in this range (11, 12). Further investigation and management of individuals with milder degrees of hyperprolactinaemia (600–2000 mIU/L) could be individualised but there is consideration for estradiol and/or cyproterone acetate dose reduction and re-evaluation. Further investigation could also be considered in individuals in whom the serum prolactin concentration is increasing over time.

## 6 Discussion

In conclusion, we have the following suggestions:

### 6.1 Should clinicians routinely monitor prolactin in trans individuals undergoing feminising GAHT?

Hyperprolactinaemia is common in trans people treated with feminising GAHT regimens containing cyproterone acetate. There is consideration for monitoring a baseline serum prolactin concentration or in those with hypogonadotropic hypogonadism at baseline. However, given that the degree of hyperprolactinaemia is often mild, we suggest against routine prolactin monitoring following initiation of GAHT. Instead, we recommend monitoring only in individuals with symptoms suggestive of hyperprolactinaemia, noting that the risk is higher with cyproterone acetate compared to spironolactone or GnRH analogues.

### 6.2 How should clinicians approach investigation and management in trans individuals with symptomatic hyperprolactinaemia?

We suggest first confirming hyperprolactinaemia on repeat testing and exclusion of other causes of hyperprolactinaemia. In individuals with mild hyperprolactinaemia (600–2000 mIU/L), there is consideration to trial lower cyproterone acetate doses or an alternative anti-androgen such as spironolactone and re-assess. Pituitary MRI and/or anterior pituitary hormone profile should be considered in individuals with a serum prolactin >2000–3000 mIU/L (approximately 4–6 times the upper limit of the reference interval).
